# Does colorectal cancer significantly influence the assembly of gut microbial communities?

**DOI:** 10.7717/peerj.3383

**Published:** 2017-06-28

**Authors:** Lin Dai, Hedan Kou, Yao Xia, Xiujun Wen, Jianpeng Gao, Zhanshan (Sam) Ma

**Affiliations:** 1 Faculty of Science, Kunming University of Science and Technology, Kunming, China; 2 Computational Biology and Medical Ecology Lab, State Key Lab of Genetic Resources and Evolution, Kunming Institute of Zoology, The Chinese Academy of Sciences, Kunming, China; 3 College of Forestry and Landscape Architecture, South China Agricultural University, Guangzhou, China; 4 Yan’an Hospital, Gastroenterology Department, Yan’an Hospital, Kunming, China

**Keywords:** Colorectal Cancer, Gut microbial community, Neutral theory, Niche theory, Diversity

## Abstract

Colorectal cancer (CRC) is the third commonest malignant tumor. Previous studies have revealed that the composition change of the human gut microbiome, measured by community diversity, is associated with the progression of CRC. However, a further question, whether or not the mechanism of community assembly and diversity maintenance of the gut microbiome is influenced by CRC has not been addressed. To address this question, we applied Hubbell’s neutral theory for biodiversity to reanalyze the dataset from Wang et al.’s (2012) study of the gut microbiome sampled from *46* CRC patients and *56* healthy individuals. Our reanalysis presents two important findings. Firstly, our analysis demonstrated that only around 4% (*4/102*) samples (in total of both the CRC and control groups) have their species abundance distribution (SAD) satisfied the prediction of the neutral theory null model. No significant difference in the number of the samples satisfying the neutral null model was detected between the healthy individuals and CRC patients, suggesting that the nature or mechanism of community assembly and diversity maintenance of the gut microbiome is not significantly influenced by CRC. That is, the stochasticity of survival, reproduction and migration of gut microbes, as implied by the neutral theory model, does not play a significant role in shaping the community assembly and diversity maintenance. We further infer that the alternative hypothesis to the neutral null model, i.e., the deterministic *niche differentiations* should be the driving forces that shape the assembly and diversity maintenance of the gut microbiome in both the healthy individuals and CRC patients. Secondly, although CRC does not seem to influence the nature of community assembly, we postulate that it may indirectly influence the outcome (i.e., the community composition as measured by community diversity) of the community assembly, possibly by influencing niche differentiations. This postulation is supported by our second finding: the diversity of the gut microbiome in CRC patients is significantly lower than that in the healthy individuals as demonstrated by the fundamental diversity parameter (*θ*) of the neutral theory model. This second finding offers an independent confirmation of the relationship between the CRC disease and diversity of the gut microbiome, about which existing studies have presented conflicting evidences. Finally, we suggest that hybrid modeling which integrates both the neutral and niche theories should be explored in future studies to further understanding of the CRC influence on the human gut microbiome.

## Introduction

The colorectal cancer (CRC), estimated to cause more than one million new cases and 700 thousand deaths each year worldwide, is the third commonest malignant tumor (Ferlay et al., 2015). Typically, the risk factors of CRC are recognized as increasing age, heredity, obesity and unhealthy life styles, such as excessive alcohol consumption and smoking. In recent years, the implications of the gut microbial community in the progression of CRC have attracted increasing attentions from biomedical communities.

From an ecological perspective, the assemblage of total microorganisms in a specific site of human body represents a complex microbial community (i.e., microbiota) that develops in parallel with host and interacts with internal physiological environment of host and external physical environment. The well-known NIH Human Microbiome Project (HMP) was rightly aimed at identifying and characterizing microbial communities colonizing in or on the five major human body sites (i.e., oral, skin, vaginal, gut and airway), where inhabited the richest and the most diverse human microbes. Among them the gut microbiota that has the largest numbers of individuals and species compared to other body sites, which consists of about 100 trillion microorganisms and of 100 times more unique genes than the human genome (Ghaisas, Maher & Kanthasamy, 2015), is obviously the most important and has been studied most extensively.

With the exception of defined pathogens, the vast majority of the gut microbes have considerable beneficial effects to our health, such as protecting against pathogens, extracting nutrients and energy from diets and contributing to normal immune function (Lozupone et al., 2012). The change of composition and function of gut microbiota has been found to be associated not only with various disorders or diseases related to digestive tract, such as obesity (Ley et al., 2006; Le et al., 2013; White, 2016; Duca et al., 2016), inflammatory bowel disease (IBD) (Frank et al., 2007; Dicksved et al., 2008; De et al., 2012) and colorectal cancer (Arthur et al., 2012; Geng et al., 2013; Sun & Kato, 2016), but also with certain common chronic diseases including type 2 diabetes (Qin et al., 2012), cardiovascular disease (Howitt & Garrett, 2012; Mafra et al., 2013), liver cirrhosis (Qin et al., 2014) and so on, even with neurological disorders, where the “microbiome-gut-brain axis” plays a great part, such as Parkinson’s and Alzheimer’s disease (Ghaisas, Maher & Kanthasamy, 2015). The establishment and maintenance of normal and well-balanced composition and function of microbiota may be a key to prevent microbiota-related diseases and a promising target for treatment. The change of normal microbiota could be developed as a sensitive indicator for the progression of related diseases as well.

Our particular concern in this study is the relationship between the CRC and the assembly mechanism of gut microbiota. Increasingly accumulating evidences from existing studies suggest that not only the individual microbial species, but also the gut microbiota, particularly its metabolome, contribute to the etiology of CRC (Louis, Hold & Flint, 2014; Sun & Kato, 2016). Metagenomics promoted by the development of the Next Generation Sequencing (NGS) has made it possible to study the total microbes as an ecological community effectively, and a number of novel results have been revealed by such studies. Wang et al. (2012) described different gut microbiota structure patterns in the CRC patients compared to healthy individuals and found a significant reduction of butyrate-producing bacteria in the gut microbiota of CRC patients. Geng et al. (2013) demonstrated that the excessive *Roseburia* at tumor sites and the excessive *Microbacterium* and *Anoxybacillus* bacteria at the adjacent non-malignant site were closely associated with Chinese CRC patients. Zackular et al. (2014) studied the gut microbiota of CRC patients and healthy individuals and developed an improved screening tool for CRC that integrated the microbiota data and known clinical risk factors. Significantly reduced microbial diversity and increased richness index were detected in the feces of CRC patients compared with those of healthy individuals and in the cancer tissue compared with the paired normal tissue (via analyses of community diversity/richness indices based on 16s rRNA sequencing data of gut microbiota), yet, some other studies failed to detect significant differences in these alpha diversity indices (Sun & Kato, 2016). The inconsistency in existing studies makes the question of what forces are controlling the gut microbial community assembly and whether they would be changed by CRC, the focus of the present study, certainly worth investigating. Further evidence to resolve the existing inconsistency is also worthy of pursuing.

Even though the change of gut microbiota has been recognized being closely related to the progression of CRC, to the best of our knowledge, the ecological question of whether the force that shapes gut microbiota is associated with CRC is still poorly understood. The gut microbiota is essentially a complex ecological community that has little fundamental difference with other ecological communities in nature such as forests and lakes. Therefore it should be submissive to the analyses governed by ecological theories traditionally developed in the macro-ecology of plants and animals, where one of the core topics is the mechanism of community assembly and diversity maintenance, which is also the choice of our analysis to shed light on the influence of CRC disease on the gut microbiome. The classical two major theoretical models describing the mechanism are the *niche theory* underlining the deterministic interactions between species, individuals and environment, as well as the *neutral theory* emphasizing the stochastic forces associated with factors such as immigration, birth/death and speciation (Ofiteru et al., 2010). The *neutral theory* may best be characterized by the assumption that “*All individuals within a particular trophic level have the same chances of reproduction and death regardless of their species identity”* (Hubbell, 2001; Li & Ma, 2016). It combines neutrality, stochasticity, sampling and dispersal and presents a *null* model to test the mechanism of community assembly and biodiversity maintenance in ecological communities (Hubbell, 2001; Etienne, 2005; Alonso, Etienne & Mckane, 2006). As a null model, regardless of the success or failure of neutral theory model to the community data, the theory offers a tool to accept or reject specific hypothesis regarding the mechanism underlying community assembly and diversity maintenance (Li & Ma, 2016). In addition, neutral theory provides an alternative approach to assessing biodiversity to the traditional diversity indexes (Alonso, Etienne & Mckane, 2006), which allows us to draw evidence helpful for resolving the conflicting results in existing studies regarding the relationship between community diversity and CRC progression. For these merits, we apply the neutral theory to comparatively explore the mechanism of community assembly and diversity maintenance in the gut microbiota in both CRC patients and healthy individuals.

Methodologically, in this study, we used two sampling formulae (models) that are widely used to test the neutrality and proposed by Ewens (1972) and Etienne (2005), respectively, to reanalyze the dataset originally reported by Wang et al. (2012). Briefly, in their study, 46 CRC patients and 56 healthy individuals were recruited and the fecal bacterial diversity of the samples from all 102 subjects were profiled by 454 pyrosequencing of the V3 region of the 16s rRNA gene. The raw data is available at the Short Reads Archive (accession number SRP005150). With the two formulae and dataset for testing the fitting of the neutral theory model to gut microbiome samples from both the healthy and diseased individuals, we aim to accomplish two objectives. First, we test the suitability of the neutral theory model to the gut microbiome of the healthy and diseased individuals, respectively. If the datasets fail to pass the neutrality test, we accept the alternative hypothesis that the *niche differentiation* is the driving force that shapes the assembly of gut microbial community. If it does pass the neutrality test, we accept the premises that all individuals in the gut microbiome are ‘born’ to have equal opportunities “to prosper or fail” regardless of their species identity, that the community is assembled randomly essentially, and the observed biodiversity is shaped by the randomness (stochasticity) associated with microbial survival, reproduction, and migration in gut microbiome. Second, regardless of the success of failure of the neutrality test, by using the biodiversity parameter from the neutral theory model, we hope to shed light on resolving the issue whether or not CRC progression is associated with the composition change of the gut microbiome, of which existing studies have presented conflicting evidences.

## Materials and Methods

### Dataset description

Forty-six gut microbiome samples from patients with CRC and fifty-six gut microbiome samples from healthy individuals were pyro-sequenced successfully by Wang et al. (2012), and the raw data generated by their sequencing operations were downloaded from the Short Reads Archive (accession number SRP005150). We recalculated the OTU (Operational Taxonomic Units) tables with 98% similarity cutoff threshold from the raw data using the *Mothur* software pipeline (Schloss et al., 2009). We picked 4,868 OTUs in total with a range of 12–266 OTUs *per* sample. Our recalculated dataset was then divided into 2 groups: the CRC group (the samples from CRC patients) and the control group (the samples from healthy individuals).

### The computational procedure for the neutrality test

We used two sampling formulae proposed by Ewens (1972) and Etienne (2005) respectively to test the fitting of neutral theory model to the gut microbiome dataset. The Ewens formula was originally developed for testing the neutrality of gene mutation in molecular biology by Ewens (1972) and later adapted for testing the neutral theory of biodiversity (Hubbell, 2001). The formula describes the probability of species abundance distribution without dispersal limitation. The Etienne (2005) formula added the dispersal limitation to Ewens formula and should perform better if the dispersal limitation plays a significant role in shaping community. Dispersal limitation is controlled by parameter *m*, which captures the probability of a randomly chosen individual in a local community is replaced by an offspring of a randomly chosen individual from the metacommunity.

To evaluate which of the two formulae is more suited for the gut microbiome dataset, we compared the *log-likelihoods* computed from both formulae. The better one (with larger likelihood value) was then used to test if the observed microbial community in samples satisfied the neutral theory by comparing the *log-likelihoods* of the observed microbial community and neural-theoretic (artificially simulated) microbial community. We also expect that the possible difference between Ewens and Etienne *log-likelihoods* may reveal the existence or lack of *dispersal limitation* in the gut microbial community.

The Ewens (1972) sampling formula was originally proposed to describe the probability distribution of alleles in gene mutations and later introduced into ecology by Hubbell (2001) to compute the likelihood of the presence of an ecological community consisting of *S* species with abundance of *n*_1_, *n*_2…_*n*_*s*_ and to measure the compliance of neutral theory prediction. The formula has the following form:

(1) \begin{eqnarray*}\Pr({n}_{1},{n}_{2},\ldots ,{n}_{s}{|}\theta ,J)= \frac{J{!}{\theta }^{s}}{{1}^{{\phi }_{1}}{2}^{{\phi }_{2}}\ldots {J}^{{\phi }_{J}}{\phi }_{1}{!}{\phi }_{2}{!}\ldots {\phi }_{J}{!}\prod _{K=1}^{J}(\theta +K-1)} \end{eqnarray*}

where *K* is counter variable ranging from 1 to *J*, *J* is the number of individuals in the community, *S* is the number of species in the community, *θ* is biodiversity parameter, *n*_*i*_ is the abundance of species *i*, and *ϕ*_*a*_ is the number of species with abundance *a*. This formula does not include the effect of dispersal limitation, which implies that the immigration rate *m* =1.

Etienne (2005) proposed a new formula for testing the neutral theory for biodiversity that considers the dispersal limitation explicitly (i.e., *m* < 1). It has the following form:

(2) \begin{eqnarray*}P(D{|}\theta ,m,J)= \frac{J{!}}{\prod _{i=1}^{S}{n}_{i}\prod _{j=1}^{J}{\phi }_{j}{!}} \frac{{\theta }^{S}}{(I)_{J}} \sum _{A=S}^{J}K(D,A) \frac{{I}^{A}}{(\theta )_{A}} \end{eqnarray*}

where *m* is defined as:

(3) \begin{eqnarray*}m= \frac{I}{I+j-1} \end{eqnarray*}

and *K*(*D*, *A*) is defined as:

(4) \begin{eqnarray*}K(D,A)=\sum _{\{{a}_{1},{a}_{2},\ldots ,{a}_{s}{|}\sum _{i=1}^{S}{a}_{i}=A\}}\prod _{i=1}^{S} \frac{{\bar {S}}_{({n}_{i},{a}_{i})}{\bar {S}}_{({a}_{1},1)}}{{\bar {S}}_{({n}_{i},1)}} .\end{eqnarray*}

We compared the performance (measured in *D*) of two formulae on the dataset to detect the possible effect of dispersal limitation using the following log-likelihood ratio test,

(5) \begin{eqnarray*}D=-2\ln \nolimits \left( \frac{{L}_{0}}{{L}_{1}} \right) =-2[\ln \nolimits ({L}_{0})-\ln \nolimits ({L}_{1})]\end{eqnarray*}

where, *L*_0_ is the null model and *L*_1_ isthe alternative model, *D* is the deviation that is twice the difference between the *log-likelihoods* of the two formulae. The *p-value* computed follows a *X*^2^-distribution with the degree of freedom being one.

We used the *exact neutrality test* method (Etienne, 2007) to test the neutrality of community (i.e., the statistical fitness of the neutral theory model to the dataset). In brief, we first apply the *maximum likelihood estimation* (MLE) method to estimate the parameters of the neutral model from the observed samples. The *de facto* standard *R* package UNTB for testing the neutral theory (Hankin, 2007) was utilized to perform the MLE. Second, for each sample, we simulated 100 artificial communities (datasets) using parameters (*θ*, *I*, *J*) estimated from observed samples and then used Etienne’s formula to calculate the likelihood for each artificial dataset, namely *P*_*s*_. Finally, we compared the mean value of the likelihoods (*P*_*s*_) of 100 artificial datasets for each sample and the likelihood (*P*) of corresponding observed sample using the Chi-squared test under the null hypothesis that there is no significant difference between the probability from observed community and the values computed from the artificial data sets. If no significant difference between *P*_*s*_ and *P*_0_ was detected, the community would be considered neutral.

## Results and Discussion

### The comparison of Ewens and Etienne sampling formulae

Detailed results of the gut microbiome calculated with Ewens and Etienne formulae were exhibited in the online supplementary Tables S1 and S2, respectively. The comparison of the performance of two methods shows that Ewens and Etienne formulae made no significant difference in terms of the *log-likelihood* (*p* > 0.05) in all the samples we tested. In another word, both methods performed equally well with the dataset. Given that one sample failed to compute the result using Etienne’s formula because of too few species, the Ewens formula was used to test the neutrality in the following analysis.

### Ewens’ neutrality exact test and comparison of the diversity parameter (*θ*)

**Figure 1:**
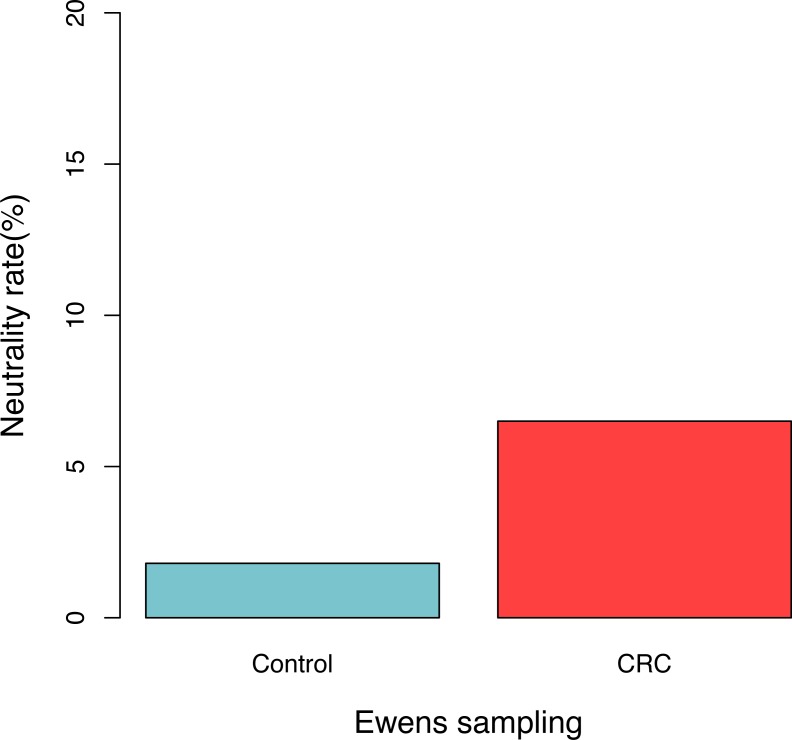
The percentage of samples that passed the neutrality test in the control (healthy individuals) and CRC (patients), respectively. No significant difference was detected between both the groups based on Fisher’s exact test (*p* = 0.324).

**Figure 2:**
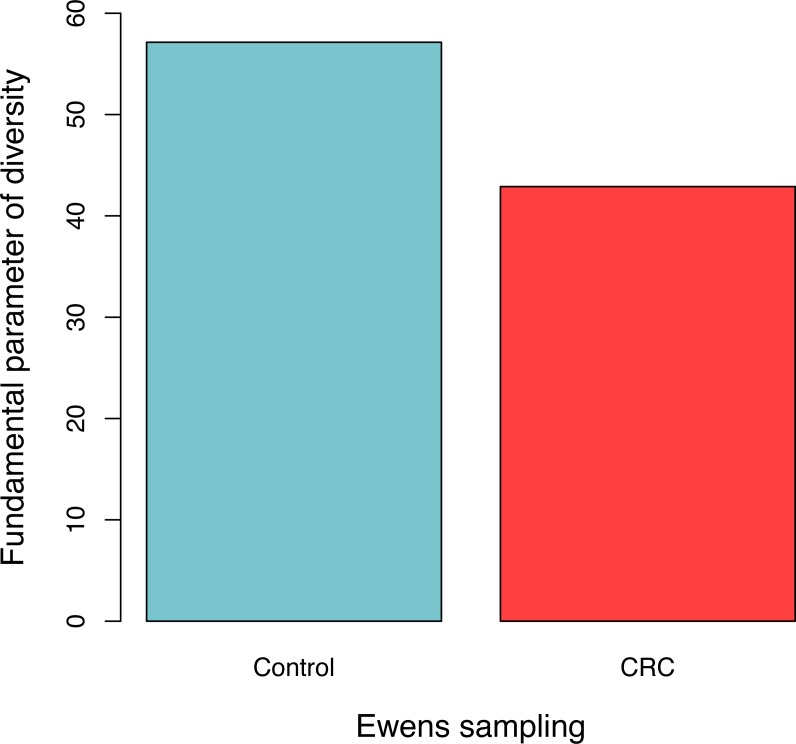
The average fundamental diversity parameter (*θ*) in the control (healthy individuals) and CRC (patients), respectively. There is significant difference between both the groups based on Student’s *t*-test (*p* < 0.01)

For each sample, the likelihood (*P*_0_) of observed dataset and the average likelihood (*P*_*s*_) of corresponding 100 artificially simulated datasets were compared using the *log-likelihood ratio* test. In total, approximately 3.9% (4/102) samples passed the *exact neutrality test*. The test results of the samples that passed the neutrality test are listed in Table 1, and the results of the rest samples are listed in the online supplementary Tables. In the CRC group, 6.5% (3/46) satisfied the neutral prediction, and in the control (healthy) group 1.8% (1/56) samples satisfied. The result of the positive rates of neutrality tests from both the healthy (control) and CRC groups is also displayed in Fig. 1. No significant difference between both the groups was detected statistically (*p* = 0.324). The average diversity parameter *θ* of each group was calculated and displayed in Fig. 2. The average *θ* value of CRC group is 42.89 and the average *θ* value of the control group is 57.13. The CRC group exhibited significantly higher *θ* value than the control group (*p* < 0.01).

**Table 1:** The five gut microbiome samples that passed the exact test of neutrality^a^.

Group	Ewens sampling formula					Etienne sampling formula		
	Sample ID	*J*	*S*	*θ*	*p*-value	*θ*	*m*	*p*-value
Control	H39	479	57	16.654	0.1306	16.64	0.99999	0.9555
	H08	919	70	17.452	0.0425	17.43	0.999233	0.0615
CRC	C39	777	66	17.056	0.3898	17.05	0.999977	0.5717
	C51	328	45	13.899	0.2127	13.9	0.999658	0.1157
	C21	77	12	3.745	0.1788	–	–	–

**Notes.**

a J, the total number of reads in the sample; *S*, the number of species in the sample; *θ*, fundamental parameter of biodiversity; *m*, immigration probability; *p*-value, the values of the likelihood ratios.

## Discussion

The presence of certain bacteria in the human gut has been shown to be associated with CRC. For example, an opportunistic pathogen, *Streptococcus bovis* was demonstrated being related with CRC using both antibody assay and fecal culture (Sun & Kato, 2016). A meta-analysis conducted by Wu et al. (2013) reported *Helicobacter pylori* infection increases the risk of CRC. Besides specific pathogens, the entire gut microbiota can also influence the progression of CRC by influencing the regular metabolome. The assembly, maintenance and dynamics of the gut microbiota should have far reaching influences on the host, in which metabolites should play a critical role. The bacteria inhabiting in gut and the mucosa of gut are exposed to the various and vast metabolite at any moment, which makes it susceptible to bacterial inflammation because of the immune defense responses of the host. The animal experiment has shown that gut microbiota is a target of inflammation that affects the progression of CRC (Louis, Hold & Flint, 2014). Tissue-associated macrophages and certain *T* cells, which exert effects *via* direct contact or *via* cytokine and/or chemokine signaling, have the potential to promote the progression of CRC (Louis, Hold & Flint, 2014). These studies suggest that it is important to draw a big picture for describing the composition of the gut microbiota of CRC patients and compare it with the normal gut microbiota of the healthy individuals, through which not only the specific opportunistic pathogens could be identified, but also the global view of the change of microbiota could be determined. Although the traditional culture-based method used to study microbiota was a gold standard to confirm the composition and function of microbiota, they are not only low throughput and inefficient but also incapable to detect majority of the gut microbes. The invention of NGS-based (Next Generation Sequencing) metagenomic technology makes it possible to discover the global-scale knowledge of microbiota, which is culture-independent and capable to detect nearly any microbes beyond bacteria and even including virus, plasmids, and bacteriophages.

A number of studies have demonstrated the changed compositions of gut microbiota in CRC patients compared with those in healthy individuals. Specific changes of the structures and patterns of gut microbiota related to the disease, particularly the relative abundances of certain significant bacteria have been reported (Wang et al., 2012; Geng et al., 2013; Zackular et al., 2014; Sun & Kato, 2016). For example, in the study by Wang et al. (2012), of which we obtained the data for this study, although no statistically significant differences were found for diversity index and evenness index, 19 specifically increased OTUs were found in the healthy individuals and 29 different OTUs were found enriched in the CRC patients. Our reanalysis of Wang et al. (2012) dataset shows that the gut microbiota of healthy individual has significant higher diversity than CRC patients, as revealed by the diversity parameter *θ* of the neutral theory model (see Fig. 2). Our result is consistent with some of the studies reviewed by Sun & Kato (2016), although some of the other studies reviewed by Sun & Kato (2016) showed no significant differences.

All the results regarding the diversity-CRC relationship were obtained by computing the diversity indexes. Our result is the first attempt to evaluate the relationship with an alternative approach—the biodiversity parameter (*θ*) of the neutral theory model. Since the alternative parameter (*θ*) is derived based on the full information from SAD (Species Abundance Distribution) of community, it should be more reliable than the traditionally used diversity indexes such as Shannon index and Simpson index. This exploration of diversity-CRC relationship with the biodiversity parameter of the neutral theory constitutes the second objective of this study.

The lack of significant difference between Ewens’ and Etienne’s formula in the performance when applied to analyze dataset suggests that there may be little dispersal limitation in the gut microbiome. This is consistent with the traditional view of “*Everything is everywhere, but the environment selects*” first proposed by Baas-Becking (1934). That is, the microbes in the human gut are free to disperse within gut in general.

The primary objective of this study is to test the hypothesis whether or not the CRC disease may significantly influence the underlying assembly mechanism of the gut microbiome. The *null* hypothesis is that the neutral theory model (i.e., the zero-sum multinomial distribution) fits to either or both of the observed SAD of the gut microbiome in the healthy and diseased individuals, respectively. If the null model of neutral theory fits the SAD of gut microbiota, then we conclude that the neutral forces (stochasticity in survival, reproduction and migration) govern the assembly of the microbiota. Otherwise (i.e., the null model fails to fit), we conclude that the alternative hypothesis—*niche differentiation* is the underling forces that drive the assembly of the gut microbiota. In other words, every species in the community has its own ecological niche, deterministically shaped by the interaction between the microbial species and its environment (the host gut in our case).

Our results from testing the neutral theory model indicates that in either of the healthy and CRC groups (treatments), the null model is rejected in more than 95% of the cases. This indicates that in both the healthy and disease treatments, the niche theory prevails. That is, niche differentiations, rather than the neutral forces, shape the assembly of the gut microbiota in both the treatments. As to the effect of CRC disease, although it may indeed influences the niche differentiation, it should have no significant influence on the assembly mechanism of the gut microbiota. In other words, regardless of the presence of CRC disease, the gut microbiota is assembled according to the rules set by deterministic niche differentiations and the stochastic neutral forces plays no significant role. This, of course, does not mean that the CRC disease may not exert any effect on the gut microbiota at all. For example, CRC may significantly affect the process of niche differentiation, and consequently may indirectly affect the outcome of community assembly. This explains the difference in the biodiversity parameter (*θ*) we observed between the healthy and CRC treatments.

To the best of our knowledge, our study is the first attempt to explore the mechanism of the assembly and diversity maintenance of gut microbiota in CRC patients and to make comparisons between the healthy and diseased treatments. The exploration successfully generated three findings or hypotheses: (i) Niche differentiations, rather than neutral forces govern the assembly of gut microbiota, and CRC disease does not change the nature of the assembly mechanisms (rules). (ii) We postulate that CRC disease may indirectly affect the assembly outcome of gut microbiome by influencing the niche differentiation, and this explains the significant difference in the biodiversity parameter (*θ*) between the healthy and diseased treatments displayed in our study. (iii) Gut microbes may not be dispersal-limited, which is consistent with the traditional view of “everything is everywhere” in microbial biogeography.

Finally, it is noted that our study has two limitations. First, although SAD is still the most generally used model for describing the community, and SAD-fitting (including our fitting the neutral theory model) can be harnessed to infer the forces that shape the community, it has certain limitations (Purves & Pacala, 2005; McGill et al., 2007). For example, it is difficult to differentiate between the contributions of sampling effects and other more ecologically based effects (McGill et al., 2007). Future studies should make efforts to combine SAD with other essential information, such as identity of species and phylogenetic information, to build more sophisticated models for better understanding of the assembly mechanism of the human microbiome related to disease. Second, it should be noted that in above discussion, the neutral and niche theories were regarded as a dichotomy of the null and alternative hypothesis, which may not be rigorous statistically as there are multiple factors (forces) that shape the community. Furthermore, niche-neutral is a *continuum* with the traditional neutral and niche theories as two ends, and more recent hybrid models that integrate the niche and neutral mechanisms should be more realistic (Leibold & Mcpeek, 2006; Fargione, Brown & Tilman, 2003; Remi, Dulvy & Freckleton, 2009; Kalyuzhny et al., 2014; Y Xia & ZS Ma, 2017, unpublished data). Therefore, another area of future studies may focus could be hybrid modeling of the human microbiome (Y Xia & ZS Ma, 2017, unpublished data). Nevertheless, given that the traditional niche theory and neutral theory are the *two ends* of the neutral-niche continuum, our treatment of the rejection of the neutral theory null model as evidence to support the alternative niche theory in this article does not contradict the second future research area we suggest here (i.e., the hybrid modeling of the human microbiome).

##  Supplemental Information

10.7717/peerj.3383/supp-1 Table S1 The result of the neutrality test using Ewens formula ^∗^*p* > 0.05
J, the total number of reads in the sample; S, the number of species in the sample; *θ*, fundamental biodiversity; m, immigration probability; Log(L0) is the log-likelihood of the observed sample, Log(L1) is the log-likelihood predicted by the neutral model, and *q*-value and p-value are the values of the likelihood ratios.

10.7717/peerj.3383/supp-2 Table S2 The result of the neutrality test using Etienne formula ^∗^*p* > 0.05
J: the total number of reads in the sample, S, the number of species in the sample; *θ*, fundamental biodiversity; m, immigration probability, Log(L0) is the log-likelihood of the observed sample, Log(L1) is the log-likelihood predicted by the neutral model, and *q*-value and *p*-value are the values of the likelihood ratios.

## Additional Information and Declarations

### Data Availability

The following information was supplied regarding data availability: NCBI: Accession number SRP005150.
