# Supplementary material for: Does colorectal cancer significantly influence the assembly of gut microbial communities?
Source: PeerJ. 2017 Jun 28;5:e3383. doi: 10.7717/peerj.3383 (PMC5493029; doi:10.7717/peerj.3383)
Supplement: Table S1 — ∗p > 0.05 J, the total number of reads in the sample; S, the number of species in the sample; θ, fundamental biodiversity; m, immigration probability; Log(L0) is the log-likelihood of the observed sample, Log(L1) is the log-likelihood predicted by the neutral model, and q-value and p-value are the values of the likelihood ratios. [file peerj-05-3383-s001.docx]

Table S1: The result of the neutral test using Ewens formula.

| Group | *ID* | *J* | *S* | *$\theta$* | *Log(L_0_)* | *Log(L1)* | *q-value* | *p-value* |
| --- | --- | --- | --- | --- | --- | --- | --- | --- |
| Control | H01 | 1301 | 211 | 71.134 | -125.45 | -65.40 | 120.090 | 0.0000 |
|  | H02 | 1359 | 266 | 98.575 | -103.57 | -61.51 | 84.125 | 0.0000 |
|  | H03 | 1276 | 162 | 48.977 | -103.54 | -69.17 | 68.733 | 0.0000 |
|  | H04 | 1194 | 177 | 57.220 | -113.93 | -65.24 | 97.369 | 0.0000 |
|  | H05 | 1181 | 152 | 46.202 | -108.67 | -66.99 | 83.373 | 0.0000 |
|  | H06 | 1069 | 123 | 35.694 | -103.33 | -65.47 | 75.716 | 0.0000 |
|  | H07 | 1019 | 168 | 57.031 | -89.10 | -58.75 | 60.693 | 0.0000 |
|  | H08 | 919 | 70 | 17.452 | -64.21 | -62.15 | 4.113 | 0.0425 |
|  | H09 | 570 | 104 | 37.009 | -57.71 | -43.72 | 27.979 | 0.0000 |
|  | H10 | 783 | 136 | 47.300 | -72.42 | -50.57 | 43.692 | 0.0000 |
|  | H11 | 1070 | 198 | 71.198 | -96.10 | -56.31 | 79.576 | 0.0000 |
|  | H12 | 933 | 158 | 54.316 | -95.35 | -55.84 | 79.019 | 0.0000 |
|  | H13 | 1117 | 259 | 105.548 | -107.15 | -52.46 | 109.380 | 0.0000 |
|  | H14 | 644 | 129 | 48.260 | -72.59 | -44.56 | 56.052 | 0.0000 |
|  | H15 | 905 | 124 | 38.661 | -81.95 | -58.65 | 46.604 | 0.0000 |
|  | H16 | 917 | 200 | 78.585 | -94.81 | -49.83 | 89.954 | 0.0000 |
|  | H18 | 1021 | 118 | 34.295 | -80.35 | -63.95 | 32.789 | 0.0000 |
|  | H19 | 725 | 131 | 46.455 | -75.34 | -49.26 | 52.152 | 0.0000 |
|  | H20 | 1242 | 159 | 48.232 | -110.06 | -68.86 | 82.397 | 0.0000 |
|  | H21 | 1056 | 186 | 65.228 | -94.24 | -58.05 | 72.366 | 0.0000 |
|  | H22 | 966 | 156 | 52.424 | -90.70 | -56.72 | 67.973 | 0.0000 |
|  | H23 | 1049 | 168 | 56.255 | -99.34 | -60.38 | 77.929 | 0.0000 |
|  | H24 | 1306 | 237 | 84.432 | -119.22 | -62.36 | 113.715 | 0.0000 |
|  | H25 | 1021 | 184 | 65.265 | -98.84 | -56.22 | 85.231 | 0.0000 |
|  | H26 | 1069 | 121 | 34.886 | -92.57 | -65.56 | 54.020 | 0.0000 |
|  | H27 | 979 | 223 | 89.885 | -78.95 | -49.77 | 58.368 | 0.0000 |
|  | H30 | 1084 | 156 | 49.745 | -99.73 | -62.40 | 74.657 | 0.0000 |
|  | H32 | 1099 | 233 | 90.146 | -99.26 | -53.86 | 90.804 | 0.0000 |
|  | H33 | 1059 | 173 | 58.480 | -90.92 | -59.51 | 62.806 | 0.0000 |
|  | H34 | 896 | 150 | 51.269 | -83.65 | -55.56 | 56.180 | 0.0000 |
|  | H35 | 1127 | 182 | 61.201 | -95.89 | -61.86 | 68.055 | 0.0000 |
|  | H36 | 1122 | 156 | 49.002 | -104.42 | -64.15 | 80.527 | 0.0000 |
|  | H37 | 1177 | 222 | 80.647 | -100.47 | -59.28 | 82.397 | 0.0000 |
|  | H38 | 1111 | 190 | 65.678 | -95.70 | -59.77 | 71.860 | 0.0000 |
|  | H39* | 479 | 57 | 16.654 | -44.19 | -43.05 | 2.286 | 0.1306 |
|  | H40 | 1180 | 147 | 44.079 | -86.29 | -67.52 | 37.543 | 0.0000 |
|  | H41 | 976 | 187 | 68.448 | -88.50 | -54.11 | 68.773 | 0.0000 |
|  | H42 | 1211 | 135 | 38.715 | -95.32 | -70.47 | 49.696 | 0.0000 |
|  | H43 | 1364 | 251 | 90.068 | -108.00 | -63.25 | 89.494 | 0.0000 |
|  | H44 | 1347 | 195 | 62.399 | -115.89 | -68.95 | 93.879 | 0.0000 |
|  | H45 | 1159 | 183 | 60.894 | -75.10 | -62.64 | 24.916 | 0.0000 |
|  | H46 | 1044 | 183 | 64.019 | -102.19 | -57.60 | 89.175 | 0.0000 |
|  | H47 | 922 | 162 | 56.711 | -99.34 | -54.85 | 88.994 | 0.0000 |
|  | H48 | 980 | 169 | 58.630 | -98.38 | -56.22 | 84.333 | 0.0000 |
|  | H49 | 1146 | 156 | 48.557 | -97.17 | -66.03 | 62.275 | 0.0000 |
|  | H50 | 1042 | 162 | 53.496 | -99.55 | -60.10 | 78.907 | 0.0000 |
|  | H51 | 982 | 129 | 39.515 | -83.35 | -61.20 | 44.286 | 0.0000 |
|  | H52 | 981 | 74 | 18.400 | -68.96 | -64.75 | 8.432 | 0.0037 |
|  | H53 | 1184 | 183 | 60.298 | -100.38 | -64.08 | 72.594 | 0.0000 |
|  | H54 | 1349 | 182 | 56.470 | -124.70 | -71.24 | 106.922 | 0.0000 |
|  | H55 | 1140 | 189 | 64.361 | -115.72 | -60.82 | 109.798 | 0.0000 |
|  | H56 | 1344 | 239 | 84.285 | -123.75 | -63.52 | 120.459 | 0.0000 |
|  | H57 | 986 | 127 | 38.580 | -76.06 | -62.03 | 28.053 | 0.0000 |
|  | H58 | 1015 | 184 | 65.459 | -96.55 | -56.71 | 79.695 | 0.0000 |
|  | H59 | 963 | 145 | 47.164 | -85.48 | -58.64 | 53.682 | 0.0000 |
|  | H60 | 700 | 161 | 65.184 | -75.00 | -43.55 | 62.896 | 0.0000 |
| CRC | C01 | 1312 | 207 | 68.887 | -105.85 | -65.55 | 80.610 | 0.0000 |
|  | C03 | 825 | 84 | 23.205 | -64.52 | -58.23 | 12.571 | 0.0004 |
|  | C05 | 1460 | 186 | 56.344 | -112.23 | -74.25 | 75.966 | 0.0000 |
|  | C07 | 1006 | 150 | 48.583 | -81.80 | -60.37 | 42.866 | 0.0000 |
|  | C09 | 1153 | 149 | 45.362 | -108.80 | -66.70 | 84.189 | 0.0000 |
|  | C11 | 1078 | 121 | 34.772 | -77.60 | -66.30 | 22.600 | 0.0000 |
|  | C13 | 1166 | 175 | 56.886 | -72.76 | -63.77 | 17.985 | 0.0000 |
|  | C15 | 1118 | 143 | 43.342 | -83.91 | -65.92 | 35.971 | 0.0000 |
|  | C17 | 1027 | 166 | 55.824 | -90.20 | -59.43 | 61.548 | 0.0000 |
|  | C19 | 786 | 121 | 39.718 | -75.14 | -53.52 | 43.246 | 0.0000 |
|  | C21* | 77 | 12 | 3.745 | -14.68 | -13.77 | 1.807 | 0.1788 |
|  | C23 | 848 | 139 | 47.015 | -93.83 | -54.20 | 79.264 | 0.0000 |
|  | C25 | 1893 | 259 | 80.936 | -148.32 | -80.79 | 135.079 | 0.0000 |
|  | C27 | 1208 | 165 | 51.446 | -103.97 | -66.13 | 75.674 | 0.0000 |
|  | C29 | 971 | 109 | 31.305 | -76.57 | -62.93 | 27.279 | 0.0000 |
|  | C31 | 923 | 128 | 40.122 | -71.91 | -58.51 | 26.806 | 0.0000 |
|  | C33 | 1526 | 204 | 63.076 | -128.60 | -75.12 | 106.948 | 0.0000 |
|  | C35 | 726 | 83 | 23.961 | -64.30 | -54.13 | 20.347 | 0.0000 |
|  | C37 | 444 | 63 | 19.833 | -43.82 | -40.86 | 5.917 | 0.0150 |
|  | C39* | 777 | 66 | 17.056 | -57.32 | -56.95 | 0.740 | 0.3898 |
|  | C41 | 1387 | 231 | 78.888 | -128.21 | -66.63 | 123.166 | 0.0000 |
|  | C43 | 1386 | 192 | 60.264 | -110.21 | -70.50 | 79.424 | 0.0000 |
|  | C45 | 1187 | 114 | 30.892 | -99.94 | -71.55 | 56.792 | 0.0000 |
|  | C47 | 879 | 112 | 33.847 | -76.40 | -58.83 | 35.130 | 0.0000 |
|  | C49 | 1075 | 161 | 52.267 | -81.97 | -61.29 | 41.361 | 0.0000 |
|  | C51* | 328 | 45 | 13.899 | -34.17 | -34.95 | 1.553 | 0.2127 |
|  | C53 | 1072 | 170 | 56.666 | -103.03 | -60.74 | 84.570 | 0.0000 |
|  | C55 | 846 | 123 | 39.352 | -76.35 | -55.44 | 41.818 | 0.0000 |
|  | C57 | 1147 | 171 | 55.413 | -93.22 | -63.51 | 59.420 | 0.0000 |
|  | C59 | 1343 | 155 | 45.086 | -110.77 | -73.25 | 75.042 | 0.0000 |
|  | C61 | 1115 | 169 | 55.173 | -101.61 | -62.74 | 77.741 | 0.0000 |
|  | C63 | 587 | 94 | 31.371 | -57.01 | -45.55 | 22.921 | 0.0000 |
|  | C65 | 1007 | 135 | 41.719 | -82.27 | -62.10 | 40.326 | 0.0000 |
|  | C67 | 488 | 110 | 43.918 | -42.90 | -38.30 | 9.188 | 0.0024 |
|  | C69 | 783 | 118 | 38.354 | -75.14 | -53.24 | 43.806 | 0.0000 |
|  | C71 | 592 | 86 | 27.437 | -51.42 | -47.37 | 8.094 | 0.0044 |
|  | C73 | 1215 | 150 | 44.817 | -105.94 | -68.21 | 75.461 | 0.0000 |
|  | C75 | 1206 | 114 | 30.716 | -93.45 | -71.39 | 44.115 | 0.0000 |
|  | C77 | 1070 | 102 | 27.547 | -90.38 | -67.18 | 46.397 | 0.0000 |
|  | C79 | 1288 | 206 | 68.989 | -108.03 | -65.20 | 85.658 | 0.0000 |
|  | C81 | 1270 | 146 | 42.391 | -96.99 | -71.66 | 50.657 | 0.0000 |
|  | C83 | 1048 | 140 | 43.207 | -84.49 | -63.21 | 42.552 | 0.0000 |
|  | C85 | 1277 | 97 | 24.225 | -80.55 | -74.32 | 12.448 | 0.0004 |
|  | C87 | 905 | 129 | 40.924 | -69.92 | -58.06 | 23.720 | 0.0000 |
|  | C89 | 951 | 147 | 48.392 | -83.84 | -57.72 | 52.224 | 0.0000 |
|  | C93 | 988 | 143 | 45.696 | -78.27 | -60.43 | 35.667 | 0.0000 |

* *p* > 0.05

J: the total number of reads in the sample, S: the number of species in the sample, θ: fundamental biodiversity, m: immigration probability, Log(L0) is the log-likelihood of the observed sample, Log(L1) is the log-likelihood predicted by the neutral model, and q-value and p-value are the values of the likelihood ratios.
