# Supplementary material for: Does colorectal cancer significantly influence the assembly of gut microbial communities?
Source: PeerJ. 2017 Jun 28;5:e3383. doi: 10.7717/peerj.3383 (PMC5493029; doi:10.7717/peerj.3383)
Supplement: Table S2 — ∗p > 0.05 J: the total number of reads in the sample, S, the number of species in the sample; θ, fundamental biodiversity; m, immigration probability, Log(L0) is the log-likelihood of the observed sample, Log(L1) is the log-likelihood predicted by the neutral model, and q-value and p-value are the values of the likelihood ratios. [file peerj-05-3383-s002.docx]

Table S2: The result of the neutral test using Etienne formula.

| Group | *ID* | *J* | *S* | *$\theta$ * | *$\text{m}$* | *Log(L0)* | *Log(L1)* | *q-value* | *p-value* |
| --- | --- | --- | --- | --- | --- | --- | --- | --- | --- |
| Control | H01 | 1301 | 211 | 71.17 | 0.999925 | -125.45 | -66.16 | 118.587 | 0.0000 |
|  | H02 | 1359 | 266 | 98.49 | 0.999949 | -103.57 | -62.06 | 83.030 | 0.0000 |
|  | H03 | 1276 | 162 | 48.92 | 0.999972 | -103.54 | -70.12 | 66.832 | 0.0000 |
|  | H04 | 1194 | 177 | 57.19 | 0.999947 | -113.93 | -65.12 | 97.605 | 0.0000 |
|  | H05 | 1181 | 152 | 46.26 | 0.999893 | -108.68 | -67.76 | 81.830 | 0.0000 |
|  | H06 | 1069 | 123 | 35.64 | 0.999966 | -103.33 | -66.23 | 74.208 | 0.0000 |
|  | H07 | 1019 | 168 | 57.09 | 0.999984 | -89.10 | -58.83 | 60.523 | 0.0000 |
|  | H08* | 919 | 70 | 17.43 | 0.999233 | -64.21 | -62.47 | 3.496 | 0.0615 |
|  | H09 | 570 | 104 | 36.97 | 0.999985 | -57.71 | -44.17 | 27.061 | 0.0000 |
|  | H10 | 783 | 136 | 47.33 | 0.999992 | -72.42 | -51.71 | 41.406 | 0.0000 |
|  | H11 | 1070 | 198 | 71.13 | 0.999987 | -96.10 | -57.63 | 76.951 | 0.0000 |
|  | H12 | 933 | 158 | 54.35 | 0.999752 | -95.35 | -56.69 | 77.334 | 0.0000 |
|  | H13 | 1117 | 259 | 105.58 | 0.999997 | -107.15 | -52.47 | 109.363 | 0.0000 |
|  | H14 | 644 | 129 | 48.30 | 0.999994 | -72.59 | -45.10 | 54.971 | 0.0000 |
|  | H15 | 905 | 124 | 38.67 | 0.999996 | -81.95 | -59.66 | 44.586 | 0.0000 |
|  | H16 | 917 | 200 | 78.72 | 0.999734 | -94.82 | -50.89 | 87.860 | 0.0000 |
|  | H18 | 1021 | 118 | 34.41 | 0.999889 | -80.35 | -64.74 | 31.228 | 0.0000 |
|  | H19 | 725 | 131 | 46.48 | 0.999992 | -75.34 | -48.96 | 52.750 | 0.0000 |
|  | H20 | 1242 | 159 | 48.29 | 0.999986 | -110.06 | -69.95 | 80.221 | 0.0000 |
|  | H21 | 1056 | 186 | 65.26 | 0.999993 | -94.24 | -59.27 | 69.930 | 0.0000 |
|  | H22 | 966 | 156 | 52.41 | 0.999949 | -90.70 | -57.51 | 66.390 | 0.0000 |
|  | H23 | 1049 | 168 | 56.30 | 0.999991 | -99.34 | -60.45 | 77.771 | 0.0000 |
|  | H24 | 1306 | 237 | 84.49 | 0.999845 | -119.23 | -63.23 | 111.983 | 0.0000 |
|  | H25 | 1021 | 184 | 65.31 | 0.999989 | -98.84 | -57.33 | 83.017 | 0.0000 |
|  | H26 | 1069 | 121 | 34.91 | 0.995582 | -92.64 | -66.12 | 53.044 | 0.0000 |
|  | H27 | 979 | 223 | 89.88 | 0.999996 | -78.95 | -50.60 | 56.716 | 0.0000 |
|  | H30 | 1084 | 156 | 49.64 | 0.999534 | -99.74 | -64.00 | 71.481 | 0.0000 |
|  | H32 | 1099 | 233 | 90.15 | 0.999994 | -99.26 | -55.19 | 88.134 | 0.0000 |
|  | H33 | 1059 | 173 | 58.47 | 0.999994 | -90.92 | -59.92 | 61.988 | 0.0000 |
|  | H34 | 896 | 150 | 51.31 | 0.999994 | -83.65 | -55.73 | 55.829 | 0.0000 |
|  | H35 | 1127 | 182 | 61.24 | 0.999983 | -95.89 | -61.87 | 68.052 | 0.0000 |
|  | H36 | 1122 | 156 | 49.06 | 0.999894 | -104.42 | -64.53 | 79.777 | 0.0000 |
|  | H37 | 1177 | 222 | 80.61 | 0.999721 | -100.48 | -59.56 | 81.829 | 0.0000 |
|  | H38 | 1111 | 190 | 65.62 | 0.999987 | -95.70 | -60.20 | 71.001 | 0.0000 |
|  | H39* | 479 | 57 | 16.64 | 0.999990 | -44.19 | -44.19 | 0.003 | 0.9555 |
|  | H40 | 1180 | 147 | 44.06 | 0.999989 | -86.29 | -68.70 | 35.193 | 0.0000 |
|  | H41 | 976 | 187 | 68.42 | 0.999988 | -88.50 | -54.39 | 68.216 | 0.0000 |
|  | H42 | 1211 | 135 | 38.73 | 0.999983 | -95.32 | -70.85 | 48.942 | 0.0000 |
|  | H43 | 1364 | 251 | 90.03 | 0.999990 | -108.00 | -63.70 | 88.588 | 0.0000 |
|  | H44 | 1347 | 195 | 62.35 | 0.999985 | -115.89 | -69.00 | 93.771 | 0.0000 |
|  | H45 | 1159 | 183 | 60.87 | 0.999994 | -75.10 | -62.80 | 24.605 | 0.0000 |
|  | H46 | 1044 | 183 | 64.00 | 0.999804 | -102.19 | -57.99 | 88.413 | 0.0000 |
|  | H47 | 922 | 162 | 56.78 | 0.999898 | -99.35 | -55.41 | 87.874 | 0.0000 |
|  | H48 | 980 | 169 | 58.62 | 0.999908 | -98.38 | -57.09 | 82.594 | 0.0000 |
|  | H49 | 1146 | 156 | 48.46 | 0.999531 | -97.18 | -66.18 | 62.004 | 0.0000 |
|  | H50 | 1042 | 162 | 53.57 | 0.999984 | -99.55 | -61.55 | 76.013 | 0.0000 |
|  | H51 | 982 | 129 | 39.54 | 0.999995 | -83.35 | -62.06 | 42.582 | 0.0000 |
|  | H52 | 981 | 74 | 18.38 | 0.999781 | -68.96 | -65.33 | 7.272 | 0.0070 |
|  | H53 | 1184 | 183 | 60.29 | 0.999994 | -100.38 | -64.15 | 72.452 | 0.0000 |
|  | H54 | 1349 | 182 | 56.35 | 0.999549 | -124.71 | -70.98 | 107.479 | 0.0000 |
|  | H55 | 1140 | 189 | 64.34 | 0.999996 | -115.72 | -61.53 | 108.381 | 0.0000 |
|  | H56 | 1344 | 239 | 84.33 | 0.999994 | -123.75 | -63.96 | 119.578 | 0.0000 |
|  | H57 | 986 | 127 | 38.52 | 0.999989 | -76.06 | -62.21 | 27.705 | 0.0000 |
|  | H58 | 1015 | 184 | 65.52 | 0.999994 | -96.55 | -56.86 | 79.388 | 0.0000 |
|  | H59 | 963 | 145 | 47.14 | 0.999963 | -85.48 | -59.28 | 52.394 | 0.0000 |
|  | H60 | 700 | 161 | 65.14 | 0.999926 | -75.00 | -43.87 | 62.256 | 0.0000 |
| CRC | C01 | 1312 | 207 | 68.92 | 0.999994 | -105.85 | -66.67 | 78.355 | 0.0000 |
|  | C03 | 825 | 84 | 23.22 | 0.999867 | -64.52 | -58.70 | 11.633 | 0.0006 |
|  | C05 | 1460 | 186 | 56.35 | 0.999994 | -112.23 | -74.25 | 75.969 | 0.0000 |
|  | C07 | 1006 | 150 | 48.60 | 0.999992 | -81.80 | -60.55 | 42.505 | 0.0000 |
|  | C09 | 1153 | 149 | 45.53 | 0.999897 | -108.80 | -66.56 | 84.477 | 0.0000 |
|  | C11 | 1078 | 121 | 34.83 | 0.999933 | -77.60 | -66.55 | 22.109 | 0.0000 |
|  | C13 | 1166 | 175 | 56.89 | 0.999981 | -72.76 | -64.20 | 17.127 | 0.0000 |
|  | C15 | 1118 | 143 | 43.26 | 0.999963 | -83.91 | -65.83 | 36.153 | 0.0000 |
|  | C17 | 1027 | 166 | 55.87 | 0.999993 | -90.20 | -59.16 | 62.077 | 0.0000 |
|  | C19 | 786 | 121 | 39.74 | 0.999993 | -75.14 | -53.93 | 42.428 | 0.0000 |
|  | C23 | 848 | 139 | 47.07 | 0.999967 | -93.83 | -53.94 | 79.778 | 0.0000 |
|  | C25 | 1893 | 259 | 80.91 | 0.999996 | -148.32 | -82.04 | 132.577 | 0.0000 |
|  | C27 | 1208 | 165 | 51.36 | 0.999960 | -103.97 | -67.26 | 73.428 | 0.0000 |
|  | C29 | 971 | 109 | 31.25 | 0.999889 | -76.57 | -63.64 | 25.860 | 0.0000 |
|  | C31 | 923 | 128 | 40.20 | 0.999943 | -71.91 | -59.84 | 24.154 | 0.0000 |
|  | C33 | 1526 | 204 | 63.13 | 0.999993 | -128.60 | -75.31 | 106.571 | 0.0000 |
|  | C35 | 726 | 83 | 23.93 | 0.999978 | -64.30 | -54.58 | 19.435 | 0.0000 |
|  | C37 | 444 | 63 | 19.83 | 0.999611 | -43.82 | -41.19 | 5.257 | 0.0219 |
|  | C39* | 777 | 66 | 17.05 | 0.999977 | -57.32 | -57.48 | 0.320 | 0.5717 |
|  | C41 | 1387 | 231 | 78.93 | 0.999994 | -128.21 | -67.53 | 121.349 | 0.0000 |
|  | C43 | 1386 | 192 | 60.30 | 0.999833 | -110.22 | -71.21 | 78.013 | 0.0000 |
|  | C45 | 1187 | 114 | 30.85 | 0.999964 | -99.94 | -71.61 | 56.674 | 0.0000 |
|  | C47 | 879 | 112 | 33.91 | 0.999941 | -76.40 | -58.71 | 35.380 | 0.0000 |
|  | C49 | 1075 | 161 | 52.25 | 0.999794 | -81.97 | -62.30 | 39.356 | 0.0000 |
|  | C51* | 328 | 45 | 13.90 | 0.999658 | -34.17 | -35.41 | 2.475 | 0.1157 |
|  | C53 | 1072 | 170 | 56.72 | 0.999993 | -103.03 | -61.19 | 83.679 | 0.0000 |
|  | C55 | 846 | 123 | 39.39 | 0.999983 | -76.35 | -56.65 | 39.397 | 0.0000 |
|  | C57 | 1147 | 171 | 55.37 | 0.999983 | -93.22 | -64.16 | 58.109 | 0.0000 |
|  | C59 | 1343 | 155 | 45.25 | 0.999897 | -110.78 | -73.27 | 75.012 | 0.0000 |
|  | C61 | 1115 | 169 | 55.07 | 0.999961 | -101.62 | -62.65 | 77.935 | 0.0000 |
|  | C63 | 587 | 94 | 31.40 | 0.997604 | -57.03 | -46.21 | 21.636 | 0.0000 |
|  | C65 | 1007 | 135 | 41.68 | 0.999968 | -82.27 | -62.28 | 39.976 | 0.0000 |
|  | C67 | 488 | 110 | 43.89 | 0.999969 | -42.90 | -38.29 | 9.218 | 0.0024 |
|  | C69 | 783 | 118 | 38.50 | 0.999866 | -75.14 | -54.00 | 42.280 | 0.0000 |
|  | C71 | 592 | 86 | 27.47 | 0.999645 | -51.42 | -47.62 | 7.606 | 0.0058 |
|  | C73 | 1215 | 150 | 44.72 | 0.999521 | -105.95 | -69.03 | 73.832 | 0.0000 |
|  | C75 | 1206 | 114 | 30.76 | 0.999879 | -93.45 | -72.31 | 42.267 | 0.0000 |
|  | C77 | 1070 | 102 | 27.50 | 0.994648 | -90.48 | -68.07 | 44.815 | 0.0000 |
|  | C79 | 1288 | 206 | 69.07 | 0.999957 | -108.03 | -66.27 | 83.519 | 0.0000 |
|  | C81 | 1270 | 146 | 42.31 | 0.999510 | -97.00 | -72.31 | 49.381 | 0.0000 |
|  | C83 | 1048 | 140 | 43.18 | 0.999953 | -84.49 | -63.85 | 41.281 | 0.0000 |
|  | C85 | 1277 | 97 | 24.25 | 0.999976 | -80.55 | -74.89 | 11.319 | 0.0008 |
|  | C87 | 905 | 129 | 40.88 | 0.999978 | -69.92 | -58.62 | 22.605 | 0.0000 |
|  | C89 | 951 | 147 | 48.43 | 0.999993 | -83.84 | -57.89 | 51.884 | 0.0000 |
|  | C93 | 988 | 143 | 45.72 | 0.999981 | -78.27 | -60.43 | 35.675 | 0.0000 |

* *p* > 0.05

J: the total number of reads in the sample, S: the number of species in the sample, θ: fundamental biodiversity, m: immigration probability, Log(L0) is the log-likelihood of the observed sample, Log(L1) is the log-likelihood predicted by the neutral model, and q-value and p-value are the values of the likelihood ratios.
